# Patient handover between ambulance crew and healthcare professionals in Icelandic emergency departments: a qualitative study

**DOI:** 10.1186/s13049-021-00829-x

**Published:** 2021-01-28

**Authors:** Sveinbjörn Dúason, Björn Gunnarsson, Margrét Hrönn Svavarsdóttir

**Affiliations:** 1 University of Akureyri, School of Health Sciences, Norðurslóð 2, 600 Akureyri, Iceland; 2 Akureyri Hospital, Akureyri, Iceland; 3 Institute of Health Science Research, University of Akureyri, Norðurslóð 2, 600 Akureyri, Iceland

**Keywords:** Ambulance services, Emergency department, Healthcare professionals, Interdisciplinary communication, Patient handover, Qualitative research, Responsibility

## Abstract

**Background:**

Ambulance services play an important role in the healthcare system when it comes to handling accidents or acute illnesses outside of hospitals. At the time of patient handover from emergency medical technicians (EMTs) to the nurses and physicians in emergency departments (EDs), there is a risk that important information will be lost, the consequences of which may adversely affect patient well-being. The study aimed to describe healthcare professionals’ experience of patient handovers between ambulance and ED staff and to identify factors that can affect patient handover quality.

**Methods:**

The Vancouver School’s phenomenological method was used. The participants were selected using purposive sampling from a group of Icelandic EMTs, nurses, and physicians who had experience in patient handovers. Semi-structured individual interviews were conducted and were supported by an interview guide. The participants included 17 EMTs, nurses, and physicians. The process of patient handover was described from the participants’ perspectives, including examples of communication breakdown and best practices.

**Results:**

Four main themes and nine subthemes were identified. In the theme of leadership, the participants expressed that it was unclear who was responsible for the patient and when during the process the responsibility was transferred between healthcare professionals. The theme of structured framework described the communication between healthcare professionals before patient’s arrival at the ED, upon ED arrival, and a written patient report. The professional competencies theme covered the participants’ descriptions of professional competences in relation to education and training and attitudes towards other healthcare professions and patients. The collaboration theme included the importance of effective teamwork and positive learning environment.

**Conclusions:**

A lack of structured communication procedures and ambiguity about patient responsibility in patient handovers from EMTs to ED healthcare professionals may compromise patient safety. Promoting accountability, mitigating the diffusion of responsibility, and implementing uniform practices may improve patient handover practices and establish a culture of integrated patient-centered care.

## Background

In medical emergencies, patients are frequently transported by ambulances to hospital emergency departments (EDs) [1, 2]. Upon arrival, the responsibility and accountability for patient care is transferred from emergency medical technicians (EMTs) to ED staff. During this patient handover, the communication of clinical information can fail in many ways, which can threaten patient safety [3–5]. Patient handover is defined herein as “situations where the professional responsibility for some or all aspects of a patient’s diagnosis, treatment, or care is transferred to another person on a temporary or permanent basis” [5] (p. 439). This process may be particularly vulnerable at the interface between prehospital and hospital care [6–8]. The reasons for this include the dissimilar training and abilities of clinicians [9]; language barriers; and a dynamic, high-risk clinical environment frequently characterized by noise, interruptions, and time restraints [5], all of which may affect the transmission of information from one professional to another. Misunderstandings or the failure to relay information may cause delays in diagnosis and proper treatment [10–12]. Using standardized handover procedures during patient handovers may be beneficial [13–16], and improvements in this process are needed to ensure better patient safety. A common remedy has been to invoke more structured communication during patient handovers by issuing healthcare guidelines or national standards, frequently using patient handover tools, such as the communication and patient handover tool “Situation, Background, Assessment, and Recommendation” (SBAR) [17–20]. A recent review of eight heterogeneous studies in which SBAR was mostly used by physicians and/or nurses found moderate evidence of improved patient safety with SBAR implementation [17]. In addition, there may be other benefits associated with SBAR implementation, including improvements in employee satisfaction, interdisciplinary teamwork, and the quality of communication [21, 22]. Changing patient handover practices is a complicated task, though, and a greater awareness of patient handover problems and opportunities for improvement may increase the adoption of better practices [23]. The current study aimed to describe healthcare professionals’ experience of patient handovers between ambulance and ED staff and to identify factors that can affect patient handover quality.

## Methods

The Vancouver School’s phenomenological method was chosen because it can lead to a systematic explication of human experiences [24]. Table 1 shows the 12 consecutive steps of the Vancouver School method [24] and describes how they were conducted in the present study.

**Table 1:** 12 Steps of the Vancouver School method

Steps of the Vancouver School		How it was applied in this study
1.	Selecting dialogue partners (the sample).	Emergency medical technicians (EMTs) (*n* = 6: 1 EMT, 3 advanced EMTs, and 2 paramedics), nurses (*n* = 7), and physicians (*n* = 4) who were experienced in patient handovers to emergency departments (EDs) were selected as a purposive sample. Variations in work experience, level of education, and service area were ensured.
2.	Preparing the mind (silence before entering a dialogue).	Before each interview, the interviewer (the first author) examined and wrote down his preconceived ideas about the issue. Because he had been working as a paramedic for years, it was especially important to consciously push aside ideas that might influence the interviews and to open his mind to hearing something new.
3.	Participating in a dialogue (data collection).	One semi-structured, individual interview was conducted with each participant (*n* = 17). An interview guide was used to guide but not dictate the interview.
4.	Sharpened awareness of words (data analysis).	The data gathering and analysis were conducted concurrently (constant comparison). Interviews were transcribed verbatim and then read and reread to get a comprehensive impression of the whole.
5.	Beginning consideration of essences (coding).	By finding key phrases and identifying their meanings, significant statements were extracted from the transcript. Next, the themes of key statements were identified and coded. An attempt was made to continuously answer the following question: *What is the essence of what this participant is saying?*
6.	Constructing the essential structure of the phenomenon for each case (single-case constructions).	The main themes in each participant’s narrative were highlighted and grouped. The most important ones were presented in a single-case analytical-model (see an example in Fig. 1).
7.	Verifying the single-case construction with relevant participants (verification).	A single-case analytical model verification was sought from each participant, who verified that the results described their experiences correctly and that they had no further suggestions or comments.
8.	Constructing the essence of the phenomenon from all cases (metasynthesis of all case constructions).	Individual analysis models were compared internally, searching for “common threads” and differences. Then, one overall analytical model was constructed from the single-case analytical models (see Table 2). All the researchers participated in this process and made sure that the analytical model was based on the data.
9.	Comparing the essential structure with the data.	To ensure that the overall analytical model was adequate, the interviews were read again and compared with the model.
10.	Identifying the overarching theme that describes the phenomenon (interpreting the meaning of the phenomenon).	During this work, the essence of the phenomenon was shaped, and the overarching theme was put into words: *Professional patient handover through clear patient responsibility, structured communication procedures, and quality teamwork*.
11.	Verifying the essential structure (the findings) with some research participants (verification).	The overall analytical model was discussed with all the participants, all of whom agreed upon the outcome of the analysis.
12.	Writing the findings.	Care was taken to quote all the participants and to shed light on their experiences regarding the research material. This was done to increase the study’s credibility and show that the results were based on the experiences of all the participants.

According to Malterud [25], multiple researchers may strengthen the design of the study because they can supplement and contest each other. The research team included a paramedic and a physician, both of whom were experienced in emergency medical care, along with a nurse, who had expertise in qualitative research. The varying experiences and interdisciplinary knowledge of the research team ensured a wider perspective of the phenomenon and created a critical dialogue of the analysis and possible preconceptions.

### Sample and setting

The participants were recruited with purposive sampling from a group of EMTs, nurses, and physicians who were experienced in patient handover at EDs. Recruitment was limited to Iceland’s largest hospitals: National Hospital in Reykjavík (~ 100,000 ED visits annually) and Akureyri Hospital in northern Iceland (~ 15,000 ED visits annually), as well as the three ambulance services with the greatest frequency of runs.

The head of each relevant department/service area was asked to send information about the study to the pool of possible participants, and those who might be interested in participating were asked to contact the first author (SD). The number of participants was not determined beforehand. Instead, it was kept in mind that the sample should have a high level of information power, which is assessed based on whether the subject is being examined narrowly or broadly, and whether the participants have some experience with the subject [25].

Twenty-three healthcare professionals were willing to participate in the study. After 17 interviews, the data were considered enough to meet the aim of the study, and data collection was stopped. The participants were 11 men and six women, ranging from 30 to 61 years of age. All had experience with patient handovers between EMTs and EDs healthcare professionals for periods ranging from two to 32 years.

The participating EMTs had dissimilar certification levels, though: EMT, advanced EMT and paramedic. EMT certification can be obtained after 260 h of entry-level education in patient transport, while advanced EMT certification requires 350 h of additional training. Both EMT and advanced EMT education are provided in the Icelandic EMT school and are based on the U.S. National Highway Traffic Safety Administration standard curriculum. Most practicing paramedics studied for a period of nine months to one year at paramedic schools in the United States because this form of education is not provided in Iceland. Hereafter, both EMTs and paramedics will be referred to as EMTs.

National guidelines stipulate what interventions EMTs are allowed to perform. Patient handover communication in EDs is both orally and in writing in a paper journal. There are no clear guidelines or protocols for ambulance crew patient handovers in EDs. However, the SBAR communication tool was being used in both hospitals.

### Data collection

Individual semi-structured interviews with open-ended questions were used because this enables discussions with the participants more than a direct question-and-answer format [26]. The data were collected between November 2017 and March 2018. The first author conducted all the interviews at times and locations chosen by the participants, ensuring privacy and quiet for the interviews. The average interview duration was 45 min (range 26–64 min).

An interview guide based on the researchers’ clinical experiences was used. The main question was as follows: *Can you describe your experiences regarding the handover of patients transported by ambulances to hospital EDs*. This question was followed by a few open-ended questions about handover procedures, responsibilities, and communication, such as quality of information, attentiveness, information flows, written reports, and feedback. After each interview, the interview guide was reviewed, but no changes or additions were needed. To establish an overview of data, the transcribed interviews were carefully read by all the researchers, and this was a practice that was repeated throughout the analysis.

### Data analysis, interpretation, and verification

**Fig. 1:**
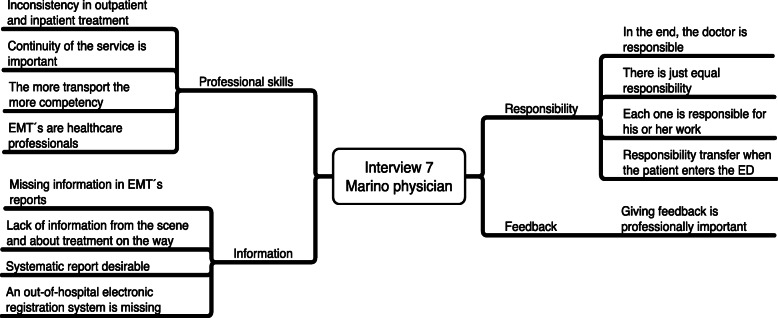
Single-case Analytical Model

The data were analyzed by a thematic analysis according to the Vancouver School [24], using NVivo 11 and MindNode 5.2.2. During data collection, the data were analyzed continuously after each interview to identify the need to go deeper into some aspects of the subject. The data were constantly revisited after each initial analysis, and each new interview was presented in a single-case analytical model (see an example in Fig. 1) and then added to the overall analytical model. Throughout the research process, the research team critically discussed the analysis and interpretation of the interviews. Codes were extracted from the data and compiled into the main themes and subthemes that sought to describe the essence of each participant’s experiences. Then, the themes were reviewed and critically discussed within the research team. Quotes were used to support the results, and each quote was identified by the participant’s profession and a pseudonym.

### Ethical considerations

The current study was conducted in accordance with the Helsinki Declaration [27]. Ethical clearance for the study was provided by the relevant institutions and was reported to the National Data Protection Authority (S8420/2017). Before the interviews, the participants received written and oral information about the study and provided written, informed consent. To enhance confidentiality, each participant was identified using a pseudonym, and any data that might reveal the participants’ identities were not disclosed. After the interviews were transcribed, the audio files were deleted.

## Results

The data analysis identified the overarching theme, “*Professional patient handover through clear patient responsibility, structured communication procedures, and quality teamwork,”* which was seen as key to handing over patients and ensuring high-quality care. Within the overarching theme, four main themes and nine subthemes were identified. The first main theme *leadership* included two subthemes in which the importance of clear *professional responsibility* for the patient and clear timepoints and locations for the *handover of responsibility* are described. The second main theme *structured framework* encompasses three subthemes: *prehospital reporting, face-to-face communication,* and *written reports*. These describe the importance of clear, formal, and structured communication before a patient’s arrival at the ED, upon ED arrival, and a written patient report for later referral. The third main theme *professional competencies* describes how professional *training* and *attitude* toward other healthcare professions, as well as the patients, can affect the quality of patient handovers. The last main theme *collaboration* addresses the importance of good interprofessional cooperation in the subtheme *team awareness,* and the importance of feedback for professional development is discussed in the subtheme *learning environment.* An overview of themes is shown in Table 2.

**Table 2:** Overall analytical model

***Overarching theme***								
Professional patient handover through clear patient responsibility, structured communication procedures and quality teamwork								
***Main themes***								
Leadership		Structured framework			Professional competencies		Collaboration	
***Subthemes***								
Professional responsibility	Handover of responsibility	Prehospital reporting	Face-to-face communication	Written reports	Training	Attitudes	Team awareness	Learning environment
***Codes***								
Shared responsibility Leading professional Responsibility for own work	Floating timepoints Floating locations Process Presence of profession	Structured Concise Accurate ED preparedness	Short and precise Formal communication Active listening Undisturbed attention	Precise Complete Valuable	Education Experience Patient volume	Disrespect Arrogance Mistrust Interest in patient cases	Teamwork Continuity of care Team members Handover procedures	Debriefing Feedback Continuing education

### Leadership

Under this main theme, two subthemes were identified. First, patients are usually managed by a hierarchy of healthcare professionals, sometimes making it unclear who is responsible for patient care and whether this responsibility is shared. Second, at times, it seems unclear where and when during the patient handover the responsibility for patient care is transferred from EMTs to the ED healthcare professionals. The participants’ views on this varied and were independent of their professions. Many participants thought it was unclear where the real responsibility lies and whether it is legal (you can be held accountable for your work) or moral (you follow your ethical and cultural principles).

#### Professional responsibility

The process of handing over patient care was seen as a responsibility shared by all healthcare professionals who were attending the patient handover. Most participants stated that one single professional would always have the greatest responsibility for the care delivered during the patient handover. Most often, this would be the person leading the actions, but education, clinical experience, and other duties could influence this. Some participants stated that this person would be the one with the highest education, meaning that this would always be a physician or a nurse. However, this assumption could become complicated when the physician had less clinical experience than the others. The participants emphasized that everyone was responsible for their own actions and that in the absence of a physician, the responsibility would be shared equally among the healthcare professionals who were present. From the nurses’ point of view, a nurse would always have some form of responsibility, even if both EMTs and physicians were present, because nurses often know the ED operating procedures best and would take care of the patient when the other healthcare professionals moved onto other tasks. In addition, interns and residents are often quite inexperienced. Another participant pointed out that it would always be the physician who would be held legally liable if something went wrong:

*It is the physician who is responsible, but we don’t have less responsibility for what we do each time. (Paul, EMT)*

#### Handover of responsibility

The participants described a lack of clarity regarding where in the patient handover process the responsibility shifted from the EMTs to the ED staff. Diverse time points and locations were mentioned, but there were no formal signals or actions that confirmed that the ED healthcare professionals had taken over responsibility from the EMTs. Many described a process that began when the patient entered the ED and ended when the EMTs had produced their report and left the patient. Many participants also noted that the responsibility shifted when the patient was out of the stretcher and on a bed, the paperwork had been handed over, and an oral report had been given to a nurse:

*In my mind, when we have moved the patient into their bed* [hospital bed]*, the report has been given, the patient is connected to their equipment* [hospital equipment] *and disconnected from our equipment and stuff, then I think they’ve got the patient. (Albert, EMT)*

The physicians were not in agreement on when in the process they had become responsible for the patient although all of them agreed that as soon as they were on site, they were in charge and responsible for the patient. Some even stated that they were responsible for the patient starting from the time they received the notification of a patient’s arrival. However, if they had not been alerted regarding the patient, some physicians were unsure who was responsible for that patient.

The nurses were more explicit on this subject and stated that they were responsible for the patient as soon as he or she had entered the ED. However, they emphasized that the EMTs should not leave the ED until a nurse or a physician had formally taken over the patient’s care:

*Somehow, as soon as he’s in the ED, it’s really the reception nurses who are responsible for the patient. (Ingrid, nurse)*

Similarly, EMTs felt responsible for the patient until other healthcare professionals had accepted the responsibility:

*We are responsible for him until someone else has taken over. (Howard, EMT)*

### Structured framework

The second main theme *structured framework* encompassed the importance of prehospital reporting before a patient’s arrival at the ED, face-to-face communication upon ED arrival, and a written patient report. This theme emphasized short, structured information, undisturbed attention, active listening, and precise written reports.

#### Prehospital reporting

Most of the participants stated that the quality and preparedness of patient handover depended greatly on the flow of information from the EMTs to the ED healthcare professionals before the patient’s arrival. The participants from all three professions described the need for more structure and improvement in the provision of prehospital reporting.

The EMTs were aware of the importance of short, targeted telecommunications that would help others prepare to receive the patient, including gathering together suitable staff.

*This depends on me, whether I want to make sure that the reception* [of the patient] *is good or not,* [...] *this is just salesmanship, if I have managed a good report to the nurse through the telecommunications and a good description and get the nurse to receive the message and repeat it, then the patient handover is of good quality. (Nathan, EMT)*

The EMTs felt that too little time was allocated to prearrival reporting. In contrast, some nurses complained about time-consuming and unstructured information, and both the nurses and the physicians noted that information was frequently inadequate and inaccurate, saying that it was more important to get a brief, concise report than a long story. Other nurses and physicians described the telecommunications as exemplary, with its information generally perceived as accurate and relevant and with the EMTs being perceived as having presented a clear picture of the patient.

#### Face-to-face communication

Face-to-face communication and first-hand oral information from the EMTs upon their arrival at the ED were seen as essential to avoid loss of information. One of the nurses explained this as follows:

*I try to get some sort of initial history from the EMT* [ … ] *because what is written on the paper is only a small fragment of what is really relevant in the handover. (Olaf, nurse)*

Again, it was obvious that concrete, short, precise information was considered necessary. The emphasis was on active listening, undisturbed attention, and eye contact to show that the listener was concentrating on what was being said. Many of the EMTs said the patient handover was often informal and that the ED staff’s attention was scattered. They complained that they were not always heard and often needed to repeat information and that it was sometimes unclear whom to report to. The physicians were also conscious of the importance of paying close attention, although they noted that it was not always done:

*This is perhaps one of the things that sometimes,* [ … ] *has gone astray or could be better, that there is silence during the handover. (Gunnar, physician)*

#### Written reports

Most of the nurses and physicians emphasized the importance of well-written, precise reports from the EMTs. These were mainly used to retain access to the information after the EMTs had left, but these reports also made the information less likely to get lost and easier to pass on, for example, between shifts. In contrast, the EMTs were skeptical about the usefulness of these reports, and some even doubted that they were ever used. Therefore, they neglected to write them, even though they were aware that they were obligated to do so. This was confirmed by both the nurses and physicians, who complained that written reports were often incomplete or missing entirely:

*In my opinion, in many cases, they could fill this out much better; there is often a lack of information about medications, blood-glucose monitoring, and, in particular, systematic information about the physical examination, the patient’s condition and treatment. Unfortunately, I often do not have the information I need. (Marino, physician)*

### Professional competences

The participants described variations of professional competence and skills in patient handovers and noted that this markedly affected the quality of the handovers. Patient volume, education, attitude, and respect were also noted as affecting the quality of patient handovers.

#### Training

Many participants claimed that differences in educational level and work experience accounted for variations in professional competence. This applied particularly to EMTs, interns, and residents and was, for example, said to be reflected in the quality of patient handovers. It was frequently mentioned for EMTs that high patient volume, but not necessarily the number of years in the profession, was related to quality of patient handovers. Paramedic education was also considered important in this respect.

The EMTs and nurses described experiences with interns who were responsible for cases that they were incapable of managing, and they emphasized the importance of experienced healthcare professionals being in charge of serious or complicated cases:

*The nurses who are at the handover are very good, but we also meet interns and residents with varying skills* [ … ]*. Some get nervous and don’t know what to do. (Albert, EMT)*

#### Attitudes

The participants also gave concerns that negative attitudes and tension between healthcare professions could affect the quality of patient handovers. This included disrespect toward other healthcare professions and toward patients. For example, some EMTs reported that their skills in assessing patients’ conditions and in providing care were sometimes questioned. They described how they felt they were looked down upon by some physicians. However, this was not common, and it was generally felt that nurses were respectful toward EMTs.

The participants emphasized the importance of respectful dialogue when in the patient’s presence. Patients who were not considered “interesting cases” or those considered of “less value” – including the homeless or alcoholics and those frequently transported to the ED – often were noted by the nurses as receiving a lower-quality of care from the EMTs, with not as much effort put into their patient handovers:

*You know some just “threw” the patient between the beds and then just disappeared. For example, old people admitted with column fractures or something like that. Then there seems to be very little interest in stopping there* [at the ED] *and giving a good report. However, when there is some exciting case, then it is possible to wait, watch, and help do things. (Laura, nurse)*

### Collaboration

Most of the participants described good cooperation between the three healthcare professions, even though this was sometimes negatively affected by heavy workloads and high stress levels in the ED. Some participants noted the benefits of using standardized procedures and delivering effective feedback for their professional development and continuity of care.

#### Team awareness

Collaboration and teamwork were emphasized, with the ultimate goal of providing the best patient care. The participants agreed that teamwork and collaboration were useful and that it was important that everyone viewed the EMTs as part of the team:

*But we are naturally working on the same goal, and this is just like one chain. (Fiona, nurse)*

However, many participants were concerned that EMTs were generally considered neither healthcare professionals nor members of the healthcare team and that this was an obstacle to good, consistent healthcare;

*What I believe is important is that this be a continuous healthcare service from the field to the hospital. This means that, immediately on the scene, information relevant to the patient’s health and treatment is collected and the treatment is adjusted to the treatment he will receive later on, so that the patient is not receiving one treatment out-of-hospital and an entirely different one at the hospital. (Marino, physician)*

The participants said that one aspect of ensuring continuity of care between prehospital and hospital care was for the EMTs and ED staff to be perceived as one team and to use uniform patient handover procedures across organizations. Some of them were not familiar with formal patient handover procedures and said no such procedures were in place. For example, most of the nurses described SBAR as an effective communication tool, but only a few EMTs where familiar with it. Although most of the participants called for structured, formal handover procedures, others claimed that standard operating procedures were mostly for beginners:

*Generally speaking,* [ … ] *standard operating procedures are good for beginners, so you learn the best way so that you have some guidelines on how to practice. But when those become routine, they are going to be in your way, because it is not always the same, and it is not always that simple. (Johann, physician)*

#### Learning environment

The participants had either no or little experience with feedback after difficult or complicated cases. However, both nurses and EMTs wished that feedback from physicians on performance and processes was a standard practice because they said it could increase their awareness of their professional competencies and decrease recurring mistakes. The EMTs desired feedback from the physicians on their performance of various tasks, including prehospital radio communication and both written and verbal reporting in the ED. They specifically expressed interest in knowing whether their reports included all the information necessary to provide seamless patient handovers. For these reasons, they saw feedback as part of continuing education and, therefore, as a way to improve their clinical competence and reduce the likelihood of mistakes in their patient handover practices:

*Feedback is very necessary and very instructive to really see, after the work done outside the hospital, what is done within the hospital and what the outcomes and fates of the individuals are.* [...] *It doesn’t necessarily have to be a meeting, but just that you can mark somewhere,* [ … ] *even if it is only a few lines of email. (Karl, EMT)*

However, many participants said that there was little time for feedback and that it was often omitted because it was not formally required. Both nurses and EMTs said that the feedback they received was mostly in form of brief remarks or complaints from their superiors. The physicians seldom received feedback, but they expressed interest in providing feedback. One physician asserted that changes in attitude and culture would be needed to enable feedback to become a standard practice.

*One is so terribly sensitive to criticism about work performance, whether one has done something incorrectly, and unconscientiously becomes defensive. Feedback culture has to be taught from basics. (Daníel, physician).*

## Discussion

In the current study, patient handover was seen as a form of teamwork where clear patient responsibility, structured patient handover communication procedures, and respect for everyone in the team and the patient were seen as important for enhancing patient safety.

Only four main themes were identified, which differed from the studies by Lawrence et al. [28] and Siemsen et al. [5]. The Lawrence et al. [28] study identified six themes related to patient handovers in the context of ED changes of shift: functions/business of ED, operations, resources, professionalism, communication, and clinical-decision processes. The Siemsen et al. [5] identified eight factors that affected the safety of patients in handovers from ambulances to hospitals and within and between hospitals: communication, information, organization, infrastructure, professionalism, responsibility, team awareness, and culture.

However, a closer look reveals many similarities. The finding from the current study of vague or unclear professional responsibilities merits some consideration. It is concerning that it is often unclear who has responsibility for the patient or where and when patient responsibility is handed over. This was not a finding in the study by Lawrence et al. [28]. However, Siemsen et al. [5] found that the giving and taking patient responsibility are often unclear or difficult, and as a result, no one clearly takes responsibility for the patient [5]. Obviously, this can lead to situations in which a patient does not receive proper treatment or is left unattended. However, previous studies have indicated that there is an unspoken understanding that the handover of responsibility occurs when the patient is transferred from the ambulance stretcher to a bed in the ED [3, 6]. This was also a common finding in the present study. Nonetheless, this issue needs to be clarified, and more-standardized patient handover procedures may help.

Many of the issues listed under communication and/or information in the other two studies [5, 28] are found in the present study under the theme of structured framework, including the importance of face-to-face communication and avoiding interruptions or distractions. The EMTs in the present study observed that too little time was spent giving prearrival information, but the nurses said that too much time was spent on this information. Even short clinical communication training might lead to better teamwork and more effective patient handovers from EMTs to ED staff [29, 30]. Unsurprisingly, the participants in the present study thought the most important aspect was to make eye contact with the recipient of the report to ensure that he or she was concentrating on it. This is in line with the results of another study that reported that EMTs value face-to-face interactions [31], which also described patient handover as exemplary when there was silence and undivided attention on the part of the recipient while the EMT was providing the patient information.

Interestingly, several nurses and physicians related the differences they observed in professional approaches to patient handover among the EMTs to the number of transports the EMTs had made and their educational level. Those with more experience and higher levels of education were perceived as performing in a more professional manner. A study from Scotland found that medical staff felt that the quality of patient handovers varied between ambulance crews [32], and the quality of patient handovers has been related to the personnel’s experiences [5]. Some authors have recommended that EMTs and ED staff receive education in structured patient handovers to improve the quality of patient handovers [32, 33].

It concerns us that some EMTs said that they were not trusted to perform their tasks or even looked down on by the ED staff, and previous research has also raised the issue of mistrust in handovers between prehospital and hospital staff [4, 12]. Patient handover in the ED requires a good understanding among different healthcare professionals about their respective roles and tasks. This is supported by research suggesting that effective teamwork is associated with improved staff satisfaction and smooth conduct of the ED [34, 35]. It has been suggested that a shared understanding can be reached with shared experiences, such as interdisciplinary training [33], and these interventions can change attitude about the importance of communication [36].

Interestingly, the present study’s narrative contained reports of disrespectful behavior toward patients, and this has also been found in other studies [3, 7, 31]. There is an abundance of evidence that abusive or disrespectful behavior is extremely dangerous in healthcare [37, 38]. Arrogant attitudes or behaviors during patient handovers may indicate a lack of teaching professionalism, ineffective leadership and disruptive workplace culture [37, 38]. We do not know how frequently this occurs, but the participants in the present study raised concerns regarding it, which indicates the need for healthcare workplaces to audit their cultures.

A number of participants in the present study commented that team awareness and acknowledging that EMTs are a part of the team is central to ensuring the continuity and coordination of care. As in the study by Siemsen et al. [5], the participants expressed that EMTs were considered by some to be neither healthcare professionals nor members of the healthcare team. This indicates prejudice and mistrust, which prevents effective teamwork and, therefore, may negatively affect patient care.

Some of the nurses and many of the participating EMTs in the present study said that they would like to get feedback from the physicians and saw it as a way to improve patient handovers and decrease recurrent mistakes but little or no time would be allotted for it. This is in line with the findings of Morrison et al. [39] where EMTs expressed a positive perception of feedback and called for receiving it from various sources more systematically. There is also a growing literature on the positive impact of using audit and feedback interventions in the ED, at least on physician performance [40, 41]. It seems that feedback is not used much by physicians in the present study, a practice which may detrimentally affect both their performance and patient outcomes.

Other issues were observed in the present study that could pose risks to patients. Disruptions and healthcare professionals’ experiences of heavy workloads could decrease their concentration regarding the transfer of liability and could increase the likelihood of misunderstandings. These findings are consistent with prior research [42].

Patient handovers have major implications for patient care, and we identified several factors that can be improved upon. We believe that the most serious one is the ambiguity in the assignment of patient responsibility. This is probably a manifestation of system failure, for example, a lack of procedures and training for the provision of safe, effective patient handovers. Another notable factor is the apparent lack of structured communication of relevant patient information in patient handovers, which has been linked to miscommunication [9, 33], and some reviews have proposed the standardization of patient handovers in this context [1, 15]. However, there appears to be a limited amount of high-quality research supporting the implementation of tools and techniques for this purpose [4]. One of the challenges in such an implementation is the boundary between ambulance services and hospitals [43]. Different organizational priorities and culture – exemplified in the current study in the implementation of SBAR communication tool in hospitals but not in the ambulance service – may stand in the way of improving in patient handover from ambulances to ED staff. Quality research on workplace culture and communication between ambulance crew and healthcare professionals in EDs is needed, to be able to focus on and better understand how to provide and accept mentorship across organizations and professions when it comes to the practice of high-quality patient handovers.

### Limitations

In the current study, we used semi-structured interviews, which enabled the participants to express their views on the topic using a wide perspective. The present study has some limitations. First, the participants were volunteers, which may have introduced bias. Second, the sample did not include healthcare professionals working at smaller locations in rural areas, part-time EMTs, or nurses and physicians in healthcare centers. In addition, the study was implemented only in one nation, so its findings lack broader generalizability. However, given the variability in the sample and the fact that the findings are supported by research from other countries, we argue that the findings are likely to be transferable beyond patient handovers in EDs in the Icelandic context.

## Conclusions

The present study’s main finding was that a lack of structured communication procedures and feedback as well as ambiguity about patient responsibility in patient handovers from EMTs to ED healthcare professionals may compromise patient safety. Promoting accountability, mitigating the diffusion of responsibility, and implementing uniform patient handover practices both within and across organizations in emergency care may improve patient handover practices and establish a culture of integrated patient-centered care. Reports of disrespectful behavior toward patients indicate the need to both audit current practices and conduct further studies.

## Data Availability

The source material supporting the conclusions of this study is available from the corresponding author upon reasonable request.

## Abbreviations

EMTs: Emergency Medical Technicians
EDs: Emergency departments
SBAR: Situation, Background, Assessment, and Recommendation

## References

[1] Dawson S, King L, Grantham H (2013). Review article: improving the hospital clinical handover between paramedics and emergency department staff in the deteriorating patient. Emerg Med Australas.

[2] Hotchin L (2011). The forgotten life savers. Health Issues.

[3] Bruce K, Suserud BO (2005). The handover process and triage of ambulance-borne patients: the experiences of emergency nurses. Nurs Crit Care.

[4] Wood K, Crouch R, Rowland E, Pope C (2015). Clinical handovers between prehospital and hospital staff: literature review. Emerg Med J.

[5] Siemsen IM, Madsen MD, Pedersen LF, Michaelsen L, Pedersen AV, Andersen HB (2012). Factors that impact on the safety of patient handovers: an interview study. Scand J Public Health.

[6] Bost N, Crilly J, Patterson E, Chaboyer W (2012). Clinical handover of patients arriving by ambulance to a hospital emergency department: a qualitative study. Int Emerg Nurs.

[7] De Lange S, Van Eeden I, Heyns T (2018). Patient handover in the emergency department: ‘How’is as important as ‘what’. Int Emerg Nurs..

[8] Burley D (2011). Better communication in the emergency department. Emerg Nurse.

[9] Di Delupis FD, Pisanelli P, Di Luccio G, Kennedy M, Tellini S, Nenci N (2014). Communication during handover in the pre-hospital/hospital interface in Italy: from evaluation to implementation of multidisciplinary training through high-fidelity simulation. Intern Emerg Med.

[10] Eggins S, Slade D. Communication in clinical handover: improving the safety and quality of the patient experience. J Public Health Res. 2015;4(3):197-9. 10.4081/jphr.2015.666 PMC4693345 26753165

[11] Foronda C, VanGraafeiland B, Quon R, Davidson P (2016). Handover and transport of critically ill children: an integrative review. Int J Nurs Stud.

[12] Knutsen GO, Fredriksen K (2013). Usage of documented pre-hospital observations in secondary care: a questionnaire study and retrospective comparison of records. Scand J Trauma Resusc Emerg Med.

[13] Bost N, Crilly J, Wallis M, Patterson E, Chaboyer W (2010). Clinical handover of patients arriving by ambulance to the emergency department–a literature review. Int Emerg Nurs..

[14] Talbot R, Bleetman A (2007). Retention of information by emergency department staff at ambulance handover: do standardised approaches work?. Emerg Med J.

[15] Jensen SM, Lippert A, Ostergaard D (2013). Handover of patients: a topical review of ambulance crew to emergency department handover. Acta Anaesthesiol Scand.

[16] Di Delupis FD, Mancini N, di Nota T, Pisanelli P (2015). Pre-hospital/emergency department handover in Italy. Intern Emerg Med.

[17] Müller M, Jürgens J, Redaèlli M, Klingberg K, Hautz WE, Stock S (2018). Impact of the communication and patient hand-off tool SBAR on patient safety: a systematic review. BMJ Open.

[18] Dayton E, Henriksen K (2007). Communication failure: basic components, contributing factors, and the call for structure. Jt Comm J Qual Patient Saf.

[19] Von Dossow V, Zwissler B (2016). Recommendations of the German Association of Anesthesiology and Intensive Care Medicine (DGAI) on structured patient handover in the perioperative setting. Anaesthesist.

[20] Haig KM, Sutton S, Whittington J (2006). SBAR: a shared mental model for improving communication between clinicians. Jt Comm J Qual Patient Saf.

[21] Andreoli A, Fancott C, Velji K, Baker GR, Solway S, Aimone E (2010). Using SBAR to communicate falls risk and management in inter-professional rehabilitation teams. Healthc Q.

[22] Edwards C, Woodard EK (2008). SBAR for maternal transports: going the extra mile. Nurs Womens Health.

[23] Clarke CM, Persaud DD (2011). Leading clinical handover improvement: a change strategy to implement best practices in the acute care setting. J Patient Saf.

[24] Halldórsdóttir S. The Vancouver school of doing phenomenology. In: Fridlund B, Hildingh C, editors. Qualitative research methods in the service of health. Lund: Studentlitteratur; 2000. p. 47–81.

[25] Malterud K, Siersma VD, Guassora AD (2016). Sample size in qualitative interview studies: guided by information power. Qual Health Res.

[26] Brinkmann S, Kvale S. Interviews: learning the craft of qualitative research interviewing. 3rd ed. Thousand Oaks Sage publications, Inc; 2015.

[27] World Medical Association (2013). World medical association declaration of Helsinki: ethical principles for medical research involving human subjects. JAMA..

[28] Lawrence RH, Tomolo AM, Garlisi AP, Aron DC (2008). Conceptualizing handover strategies at change of shift in the emergency department: a grounded theory study. BMC Health Serv Res.

[29] Scott LA, Brice JH, Baker CC, Shen P (2003). An analysis of paramedic verbal reports to physicians in the emergency department trauma room. Prehosp Emerg Care.

[30] Iedema R, Ball C, Daly B, Young J, Green T, Middleton PM (2012). Design and trial of a new ambulance-to-emergency department handover protocol: 'IMIST-AMBO'. BMJ Qual Saf.

[31] Meisel ZF, Shea JA, Peacock NJ, Dickinson ET, Paciotti B, Bhatia R (2015). Optimizing the Patient Handoff Between Emergency Medical Services and the Emergency Department. Ann Emerg Med.

[32] Thakore S, Morrison W (2001). A survey of the perceived quality of patient handover by ambulance staff in the resuscitation room. Emerg Med J.

[33] Owen C, Hemmings L, Brown T (2009). Lost in translation: maximizing handover effectiveness between paramedics and receiving staff in the emergency department. Emerg Med Australas..

[34] Ajeigbe DO, McNeese-Smith D, Leach LS, Phillips LR (2013). Nurse-physician teamwork in the emergency department: impact on perceptions of job environment, autonomy, and control over practice. J Nurs Adm.

[35] Grover E, Porter JE, Morphet J (2017). An exploration of emergency nurses’ perceptions, attitudes and experience of teamwork in the emergency department. Australas Emerg Nurs J.

[36] Aaronson EL, White BA, Black L, Brown DF, Benzer T, Castagna A (2019). Training to improve communication quality: an efficient interdisciplinary experience for emergency department clinicians. Am J Med Qual.

[37] Hickson GB, Pichert JW, Webb LE, Gabbe SG (2007). A complementary approach to promoting professionalism: identifying, measuring, and addressing unprofessional behaviors. Acad Med.

[38] Rosenstein AH, O’Daniel M (2008). A survey of the impact of disruptive behaviors and communication defects on patient safety. Jt Comm J Qual Patient Saf.

[39] Morrison L, Cassidy L, Welsford M, Chan TM (2017). Clinical performance feedback to paramedics: what they receive and what they need. AEM Education and Training.

[40] Rudiger-Sturchler M, Keller DI, Bingisser R (2010). Emergency physician intershift handover - can a dINAMO checklist speed it up and improve quality?. Swiss Med Wkly.

[41] Le Grand RR, Narvaez Y, Venkatesh AK, Fleischman W, Hall MK, Taylor RA (2015). Improving emergency physician performance using audit and feedback: a systematic review. Am J Emerg Med.

[42] Panchal AR, Gaither JB, Svirsky I, Prosser B, Stolz U, Spaite DW (2015). The impact of professionalism on transfer of care to the emergency department. J Emerg Med.

[43] Sujan MA, Chessum P, Rudd M, Fitton L, Inada-Kim M, Cooke MW (2015). Managing competing organizational priorities in clinical handover across organizational boundaries. J Health Services Res Policy.

